# Cartilage Tissue Engineering by the 3D Bioprinting of iPS Cells in a Nanocellulose/Alginate Bioink

**DOI:** 10.1038/s41598-017-00690-y

**Published:** 2017-04-06

**Authors:** Duong Nguyen, Daniel A. Hägg, Alma Forsman, Josefine Ekholm, Puwapong Nimkingratana, Camilla Brantsing, Theodoros Kalogeropoulos, Samantha Zaunz, Sebastian Concaro, Mats Brittberg, Anders Lindahl, Paul Gatenholm, Annika Enejder, Stina Simonsson

**Affiliations:** 1 3D Bioprinting Center, Dept. of Chemistry and Chemical Engineering, Chalmers University of Technology, Gothenburg, Sweden; 2 Chemical Biology, Dept. of Biology and Biological Engineering, Chalmers University of Technology, Gothenburg, Sweden; 3 Institute of Biomedicine at Sahlgrenska Academy, Department of Clinical Chemistry and Transfusion Medicine, University of Gothenburg, Gothenburg, Sweden; 4 Cartilage Repair Unit, University of Gothenburg, Region Halland Orthopaedics, Kungsbacka Hospital, Kungsbacka, Sweden; 5 Wallenberg Wood Science Center, Chalmers University of Technology, Gothenburg, Sweden

## Abstract

Cartilage lesions can progress into secondary osteoarthritis and cause severe clinical problems in numerous patients. As a prospective treatment of such lesions, human-derived induced pluripotent stem cells (iPSCs) were shown to be 3D bioprinted into cartilage mimics using a nanofibrillated cellulose (NFC) composite bioink when co-printed with irradiated human chondrocytes. Two bioinks were investigated: NFC with alginate (NFC/A) or hyaluronic acid (NFC/HA). Low proliferation and phenotypic changes away from pluripotency were seen in the case of NFC/HA. However, in the case of the 3D-bioprinted NFC/A (60/40, dry weight % ratio) constructs, pluripotency was initially maintained, and after five weeks, hyaline-like cartilaginous tissue with collagen type II expression and lacking tumorigenic Oct4 expression was observed in 3D -bioprinted NFC/A (60/40, dry weight % relation) constructs. Moreover, a marked increase in cell number within the cartilaginous tissue was detected by 2-photon fluorescence microscopy, indicating the importance of high cell densities in the pursuit of achieving good survival after printing. We conclude that NFC/A bioink is suitable for bioprinting iPSCs to support cartilage production in co-cultures with irradiated chondrocytes.

## Introduction

Three-dimensional bioprinting technology is anticipated to radically change regenerative medicine because it would enable tissues and organs to be printed on demand^1, 2^. Three-dimensional bioprinting allows the distribution of different cells and supporting biomaterials (bioink) in sophisticated ways with high spatial resolution in order to resemble the microarchitecture of different tissues. In particular, bioprinted cartilage replacements for the treatment of secondary osteoarthritis (OA) and chondral and osteochondral injuries are believed to have the potential to find early clinical translation, as the need is substantial and many materials suitable for bioprinting have been used in FDA-approved devices/systems. Putative cartilage grafts have previously been bioprinted with human mesenchymal stem cells^3, 4^. Currently, autologous chondrocyte implantation (ACI) is a cell-based procedure with a clinically acceptable outcome; however, patients are subjected to two surgical procedures, and healing is dependent on the quality and quantity of the patients’ autologous cells^5–7^. Since cartilage is immunoprivileged, heterologous cells can be used in grafting; thus, we investigated whether an established and defined human-derived induced pluripotent stem cell (iPSC) line^8^ could be bioprinted, with the advantages that such a technique reduces the need for multiple surgical procedures and offers coherent and controllable cell responses as well as unlimited supplies. Mesenchymal stem cells (MSCs) are a heterogeneous subset of stromal multipotent cells that can be isolated from bone marrow, adipose- and synovial tissue, Wharton’s jelly/umbilical cord and many other connective tissues. MSCs can differentiate into cells of the mesodermal lineage, giving rise to a range of specialized connective tissues, including bone, adipose tissue and cartilage. However, transplanted MSCs preferentially differentiate into bone, in contrast to transplanted chondrocytes, which tend to mature into cartilage^9^. Recently, the healing effects of MSCs have been explained by the ability of MSCs to interact with immune cells, leading to the modulation of inflammatory conditions such as OA. Allogenic MSCs have been used recently in combination with autologous chondrons for the treatment of cartilage lesions^10^. Here, we used both iPSCs that originated from chondrocytes and the iPS generation process to rejuvenate the cells into the blastula stage of development, which means that they are pluripotent and can give rise to any cell type in the body, including nerve cells^11^, MSCs or chondrocytes. Differentiation protocols for directing pluripotent stem cells toward the chondrogenic lineage are emerging, and the most robust protocol to date has been co-culturing with chondrocytes mitotically inactivated by irradiation, which are called iChons here and which diminish with time^12^. Newer protocols have emerged, but these include fluorescence-activated cell sorting (FACS), which of course is impossible for encapsulated cells after 3D bioprinting.

Cell viability, as well as the ability to print bioinks and maintain 3D structures long term, were investigated in two different nanofibrillated cellulose (NFC) compositions with either alginate (A) or hyaluronic acid (HA) hydrogels. NFC provides structural and mechanical support for forming the physiological mimetic environment. In the case of cartilage, the NFC mimics the bulk collagen matrix, alginate simulates proteoglycans, and hyaluronic hydrogel substitutes for the hyaluronic acid found in cartilage. Alginate and nanofibrillated cellulose, both of which are xeno-free and FDA-compliant materials, have previously been used in non-printed 3D cultures of iPSCs for expansion and differentiation towards the chondrogenic lineage^13–15^. Plant-derived NFCs have been shown to successfully maintain iPSC pluripotency and clustering into spheroids^13^, while alginate maintains iPSCs by its gentle encapsulation into microcapsules, forming clustered spheroids^14^. Hyaluronic acid-based hydrogels represent another category of FDA-compliant materials, with HA being a major component in native cartilage. These hydrogels have been shown to encapsulate iPSCs well enough for injecting into structures with desired architectures, sustaining stem cell pluripotency, and supporting differentiation in 3D^16^. These biomaterials were all found to support spatial distribution and to promote the expansion and maturation of iPSC populations in long-term cultures for up to 3–4 months^17–19^. In addition, combinations of these materials have viscoelastic properties that allow rapid prototyping via the inkjet printing of defined architectural constructs^16, 20^. To date, the combinations of these hydrogels (NFC/A and NFC/HA) have not been used to bioprint iPSCs. In this study, we 3D bioprinted iPSCs to test these hydrogel combinations for cartilage regeneration.

## Results

Protocols used to induce the differentiation of the iPSCs in the bioprinted materials were inspired by current strategies with the highest success rate, relying on co-culturing with irradiated mature chondrocytes (iChons)^8^. Growth factors combined with a 3D environment are essential for directing iPSCs towards the chondrogenic lineage. Factors such as TGFβ1, TGFβ3, GDF5, and BMP2 have been found to be crucial for the production of the important hyaline cartilage matrix components collagen type II, IX, and XI and aggrecan^21–23^.

To ensure the formation of a functional mimic of cartilage tissue, visualization of the 3D arrangement of the extracellular matrix (ECM) and living cells in native hydrated conditions is central. Hence, bright-field microscopy was complemented with nonlinear microscopy to simultaneously acquire second-harmonic generation (SHG) images of collagen and two-photon excited autofluorescence (TPEF) images of living cells in unlabeled, printed constructs^24^.

**Figure 1:**
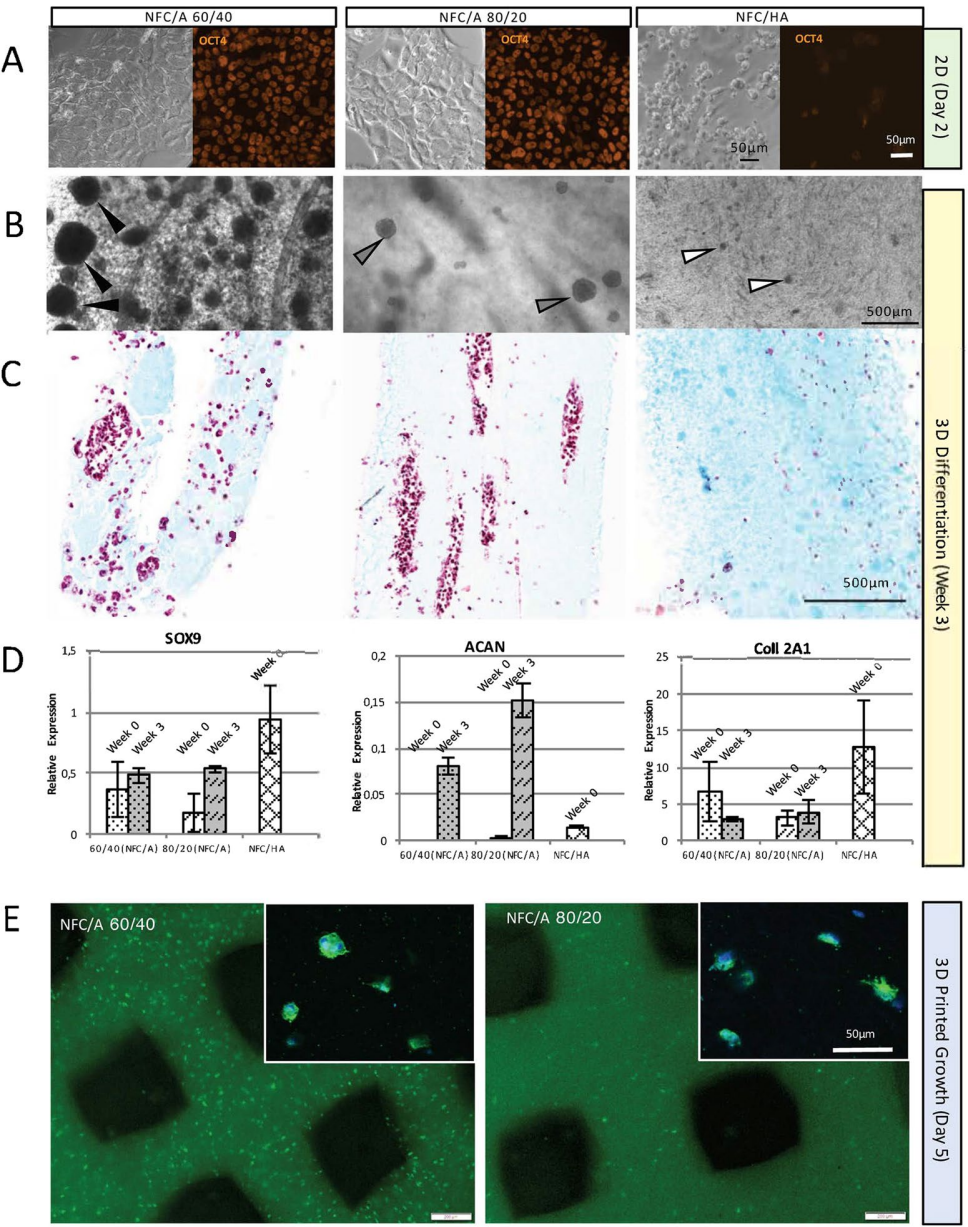
Material compatibility and cell pluripotency of different bioinks. (**A**) Bright-field and fluorescent images at day 2 of the iPSCs being in contact with the three bioink compositions: (1) NFC/A 60/40 crosslinked with 100 mM CaCl_2_ solution, (2) NFC/A 80/20 crosslinked with 100 mM CaCl_2_ solution, and (3) NFC/HA crosslinked with 0.001% H_2_O_2_ solution (the scale bars represent 50 μm). Cell morphology and Oct4-positive staining (orange) indicated the compatibility and inertness of both NFC/A treatments. However, NFC/HA treatment changed the cell morphology to be spherical with less Oct4 staining and less cells. (**B**–**D**) Encapsulation of iPSCs in bioinks for a three-week differentiation period resulted in dissimilar cell distribution and differentiation phenotypes. (**B**) The NFC/A 60/40 bioink had a greater amount of clusters with larger diameters (each black arrow points to one cluster) compared to the 80/20 bioink (each gray arrow points to a cluster). By contrast, the NFC/HA bioink had minimal cell clusters (each white arrow points to one of the low-density cell clusters) (the scale bar represents 500 μm). (**C**) Alcian blue-van Gieson-stained histological sections revealed more rounded clusters in the NFC/A 60/40 bioink compared to the elongated clusters in the NFC/A 80/20 bioink and the lack of cells and clusters in the NFC/HA bioink. (**D**) Furthermore, the three bioinks supported the phenotypic expression of SOX9, aggrecan, and collagen type 2A1. (NFC/HA samples at week 3 were lacking due to the small amount of RNA). (**E**) At day 5 after printing, the NFC/A 60/40 bioink composition was superior for cell viability compared to the NFC/A 80/20 bioink, as shown in the wide-field fluorescence images with live staining (green, live) (the scale bars represent 200 μm). Confocal images (upper right corners) show signs of cell proliferation in the NFC/A 60/40 bioink with multiple cells in a cluster (DAPI, blue; actin, green) (the scale bar represents 50 μm).

To ensure that the bioinks were compatible with the iPSC phenotypic properties, 2D monolayer culturing was conducted on the bioprinted materials with different dry weights or volume percent ratios of the structural (NFC) and cell-supporting (A or HA) components together with the crosslinking solutions (Fig. 1A and Supplementary Table 1). Brightfield microscopy images confirmed no morphological or proliferative changes for the NFC/A 60/40 wt% and NFC/A 80/20 wt% bioinks with the CaCl_2_ crosslinking solution compared to unprinted controls. Positive staining for iPSC marker OCT4 was indicative of cellular pluripotency for the cells in culture with the NFC/A bioink treatment. When in contact with the NFC/HA bioinks, the cells were rounded, with low positive OCT4 staining; hence, this material and the crosslinking conditions induced phenotypic changes of the cells away from pluripotency (Fig. 1A). The crosslinking agent, H_2_O_2_, which was used for NFC/HA, was not the cause of the reduction of OCT4 since H_2_O_2_ exposure alone did not reduce OCT4 staining (Supplementary Fig. 1).

The proliferation of iPSCs encapsulated in different bioink compositions and maintained in DEF-CS medium was examined for one week to ensure that the material was compatible with supporting 3D expansion. Observations made using bright-field microscopy demonstrated the differences in cell distribution and cluster sizes (Fig. 1B, Supplementary Table 1, and Supplementary Fig. 2). The NFC/HA bioink showed little to no proliferation of the limited cell population remaining in the construct after encapsulation. The NFC/A 80/20 wt% constructs contained an even distribution of cells that proliferated into smaller, elongated and more irregular-shaped cell clusters. The greatest amount of growth and largest spherical clusters were observed within the NFC/A 60/40 wt% constructs by bright-field microscopy as well as after sectioning and staining with Alcian blue-van Gieson (Fig. 1B and C and Supplementary Table 1).

After three weeks of growth factor-mediated differentiation (with prior maintenance of iPSCs in the hydrogels in DEF-CS for one week), RNA expression showed phenotypic increases in chondrogenic markers for all three types of bioinks (Fig. 1D). Hence, the NFC/HA bioink still seemed promising for use in further experiments if an increase in cell number could be achieved through improved proliferation and delivery. The iPSCs printed with the NFC/A 60/40 wt% bioink showed better survival than iPSCs printed in the NFC/A 80/20 wt% bioink (Fig. 1E; more live cells (stained green) are seen in the NFC/A 60/40 wt% bioink, and increased survival was observed with time, Supplementary Fig. 3). Hence, using a lower concentration (60 wt%) of NFC yielded a higher number of viable cells, although a higher concentration (80 wt%) of NFC yielded more stable and defined printed constructs (as can be seen in Fig. 1E, lower; the printed grid lines using NFC/A 60/40 did not retain the same thickness and homogeneity compared to those using 80/20, Supplementary Fig. 4).

**Figure 2:**
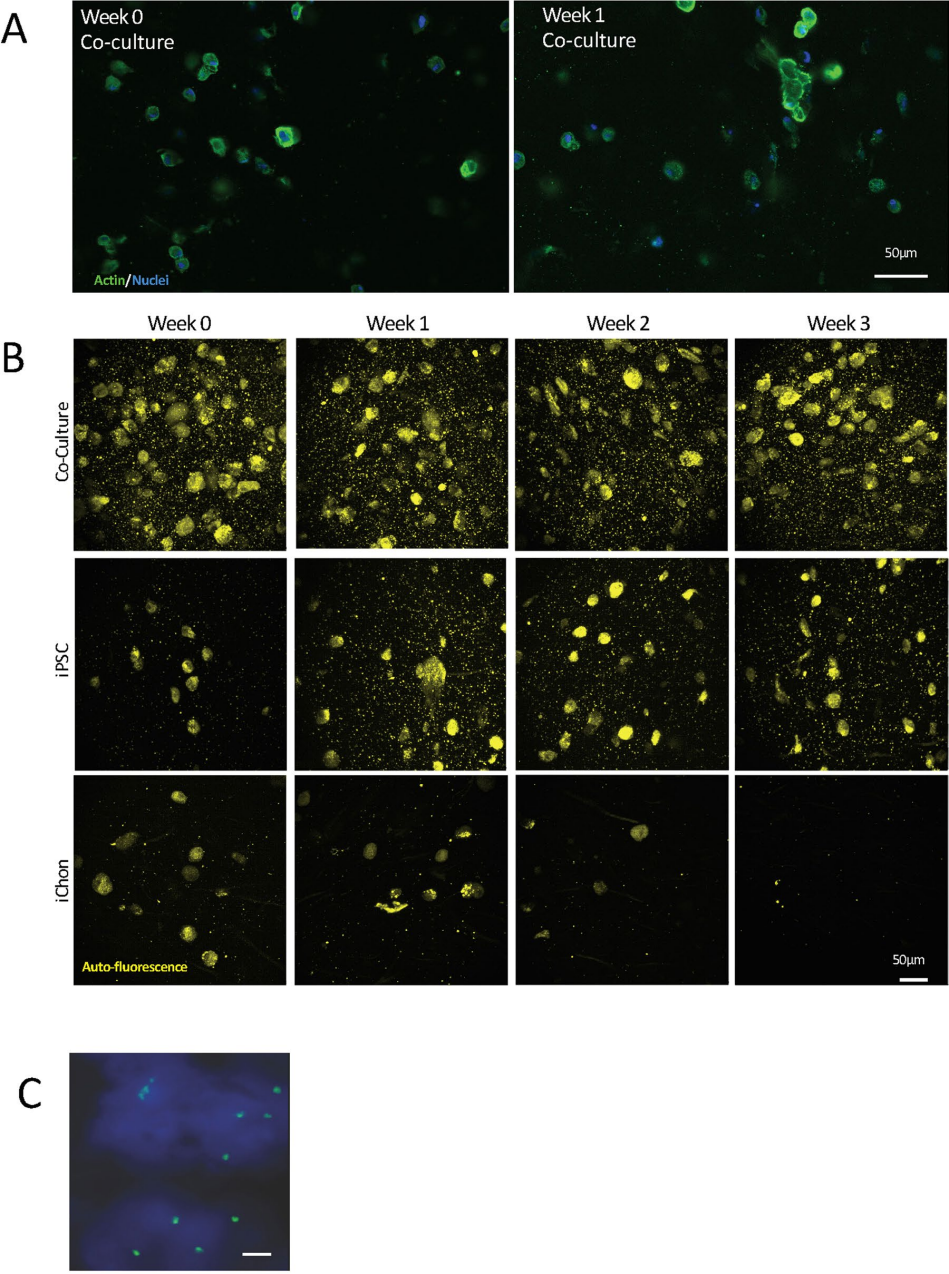
Co-culture increased iPSC density in the NFC/A 60/40 bioink after printing. (**A**) Confocal microscopy images of the NFC/A 60/40 co-culture samples at week 0 and week 1 stained for actin (green) and nuclei (blue) show cells evenly distributed with cluster formation after week 1, thus indicating proliferation (the scale bar represents 50 μm). (**B**) Label-free nonlinear microscopy (two-photon excitation fluorescence, with the autofluorescence of cells shown in yellow) images show similar distributions in the co-cultures compared to the confocal images. However, a decrease in the cell number was seen over time for the irradiated chondrocyte (iChons)-only prints as expected (co-culture prints were conducted with a 1:1 ratio of iPSC to iChons) (the scale bar represents 50 μm). (**C**) Fluorescence *in situ* hybridization (FISH) stained only the X chromosomes (X chromosomes, green; Y chromosomes, red) in the co-cultured cartilage-like tissue. Female line, iPSCs; male, iChons (the scale bar represents 10 μm).

**Figure 3:**
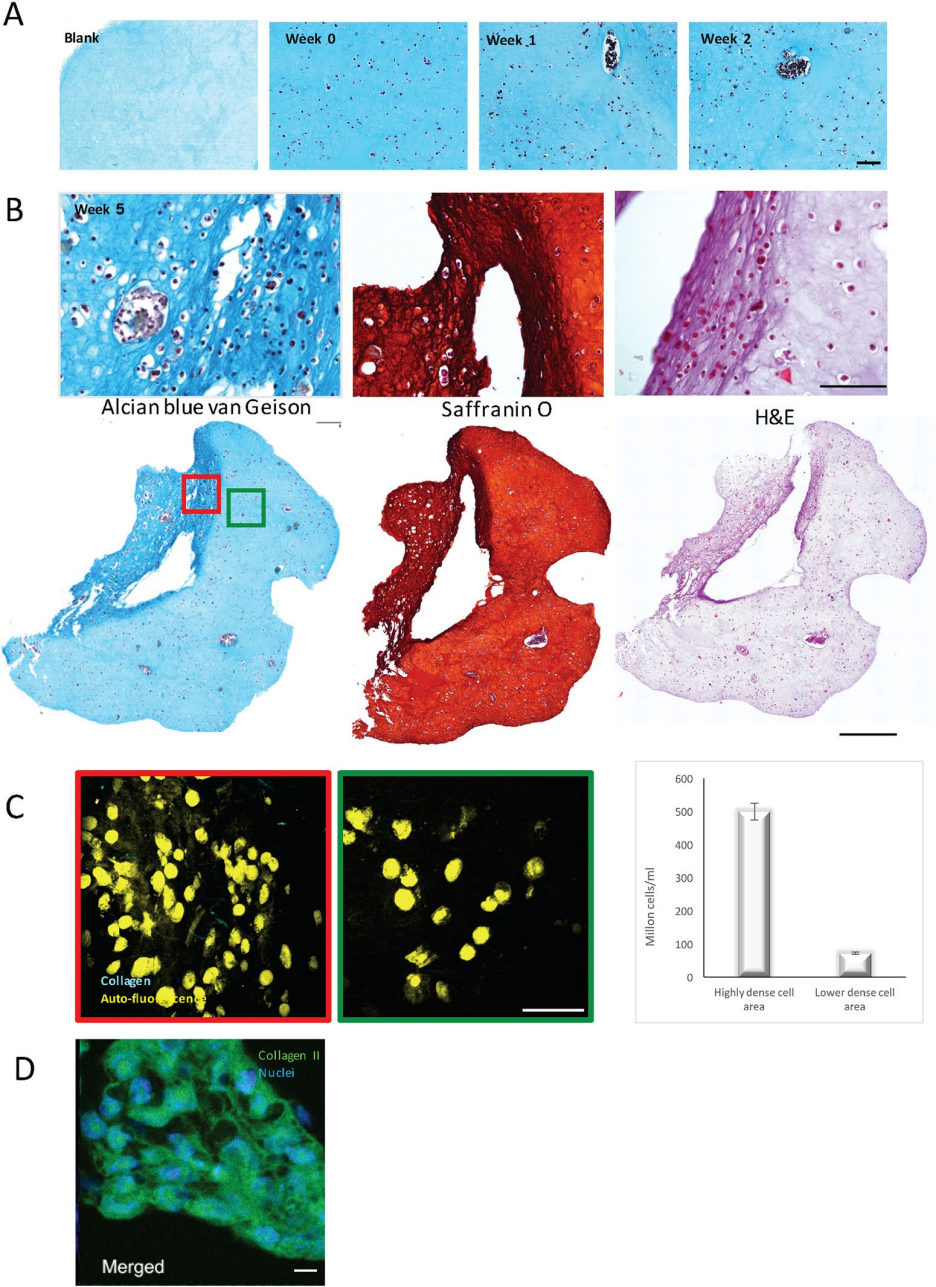
Three-dimensional-bioprinted cartilage-like tissue. (**A** and **B**) Histology sections of the 3D-bioprinted constructs. (**A**) At week 3 (blank - no cells), week 0, week 1, and week 2 of differentiation, which followed 2 weeks of proliferation in the iPSC maintenance medium (stained with Alcian blue-van Gieson for proteoglycans/glycosaminoglycans (GAGs) (blue) and nuclei (brown)) (the scale bar represents 100 μm). (**B**) The 3D-bioprinted chondrocyte-derived iPSCs (printed together with iChons, which had been diminished) at week 5 of differentiation, zoomed in (upper row) and whole section (lower row) images of sections stained for GAGs, Safranin O for cartilage (with nuclear counterstain), and hematoxylin and eosin (H&E) for extracellular matrix (with nuclear counterstain) (the scale bar represents 100 μm or 500 μm). (**C**) Label-free images of unstained sections (of areas corresponding to red and green boxes from the lower row of **B**) shows highly dense cell areas (cell autofluorescence, yellow) and collagen-like fibrils (second-harmonic generation, cyan). The highly dense cell area in the red box corresponded to higher GAG staining (the scale bar represents 50 μm). The number of cells per ml was calculated from the high-density (red square) and low-density (green square) areas. (**D**) Fluorescent image of an immunohistochemistry section (from the same 3D printed sample as **B** and **C**) stained for collagen type II (green) (with nuclear counterstain shown in blue), which shows the production of extracellular matrix collagen type II in a representative cell cluster (the scale bar represents 10 μm).

**Figure 4:**
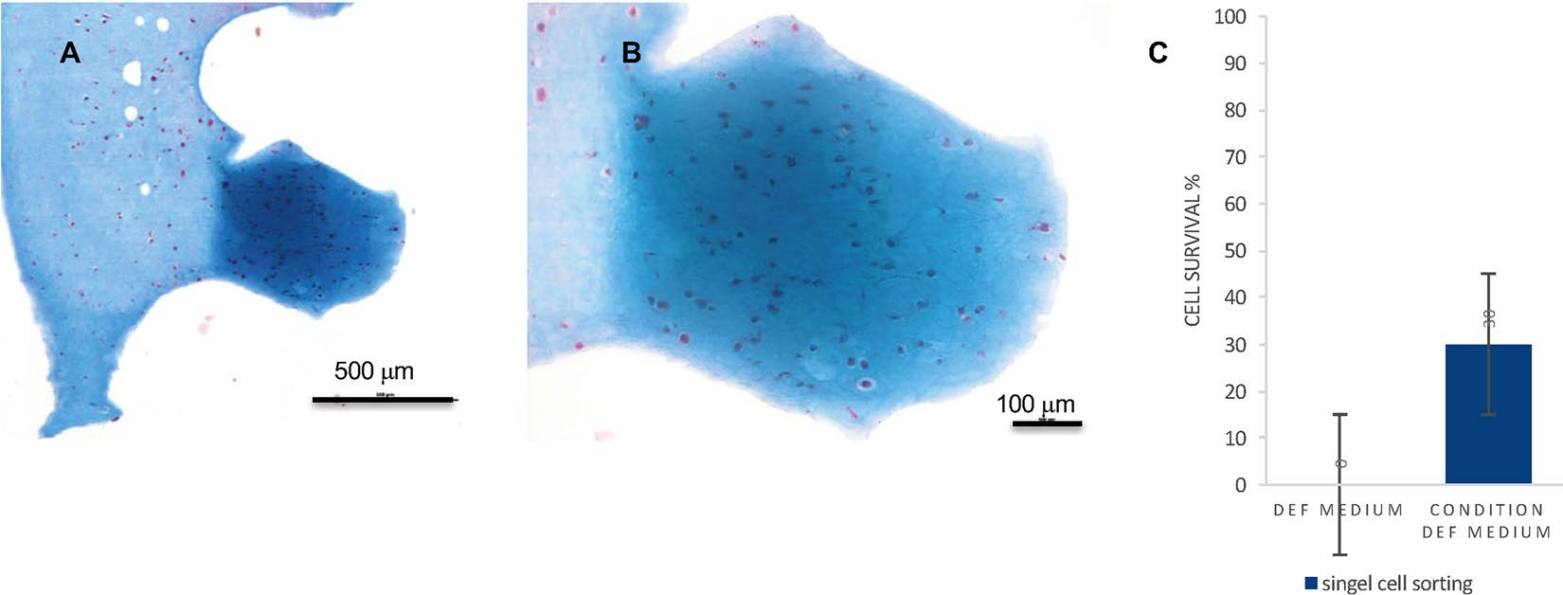
Three-dimensional-bioprinted cartilage-like tissue from iPSCs excluding iChons. (**A** and **B**) Histology sections of 3D-bioprinted constructs at week 6 (week 1 proliferation in conditioned DEF medium for the iPSCs plus 5 weeks of chondrogenic differentiation with TGFβ1, TGFβ3, GDF5 and BMP2) (stained with Alcian blue-van Gieson for proteoglycans/glycosaminoglycans (GAGs) (blue) and nuclei (brown)) (the scale bars represent 500 μm in A and 100 μm in **B**). (**C**) Cell survival of single iPSCs in the DEF medium or in the iPSC-conditioned DEF medium.

**Figure 5:**
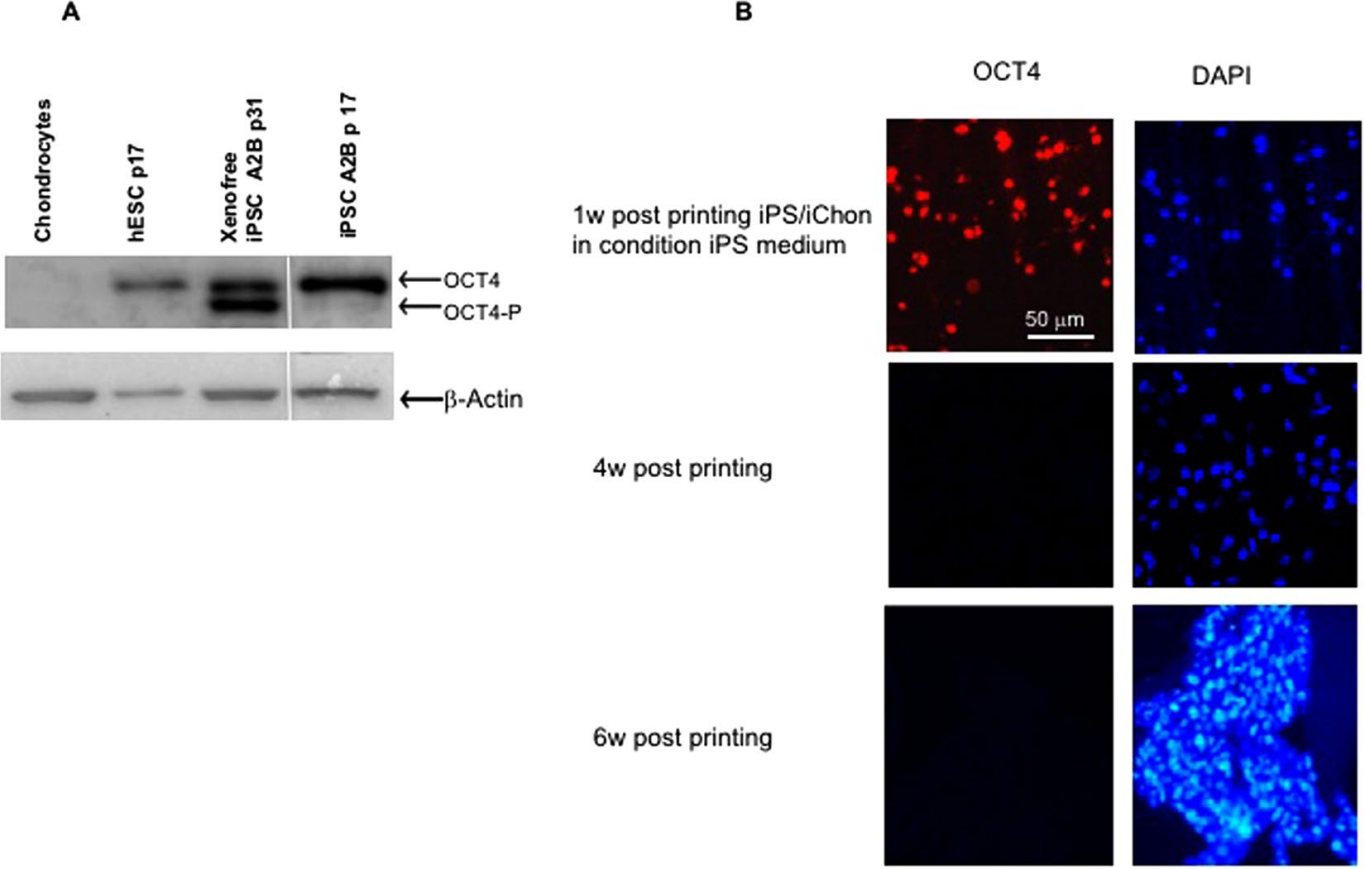
Pluripotency marker Oct4 was still expressed 1 week after 3D bioprinting the iPSCs with iChons in conditioned DEF medium, and the diminishing of Oct4 was seen after chondrogenic differentiation. (**A**) Western blot of cells before printing in the primary chondrocytes passage 1 before irradiation, in hESCs (human embryonic stem cell line SA121 passage 17), and in chondrocyte-derived iPSC line A2B in DEF xeno- and feeder-free passage 31 or DEF feeder-free culture at passage 17. No expression of pluripotency markers was detected in the chondrocyte cultures. β-Actin was used in the western blot analysis to show equal loading. (**B**) Immunohistochemistry for Oct4 and nuclei DAPI 1, 4 and 6 weeks after printing the iPSCs with iChons and maintaining the samples in iPSC medium for the first week, followed by the induction of chondrogenic differentiation (the scale bar represents 50 μm).

To direct iPSCs towards chondrocytes, we used a combination of growth factors (in short, Wnt 3a and Activin A for 3 days followed by GDF5 + BMP2 + TGFβ1 for up to five weeks) printed in the NFC/HA (Supplementary Fig. 5) or NFC/A bioink or co-cultured with irradiated chondrocytes (iChons) using the growth factors GDF5, BMP2, TGFβ1 and TGFβ3. First, the iPSCs and the iChons were printed in separate strands in an overlapping grid structure (Supplementary Fig. 6A). The production of GAGs was moderately increased in the intersections, indicating that crosstalk between chondrocytes and iPSCs was beneficial (Supplementary Fig. 6B and C). Therefore, iPSCs and iChons were mixed at a ratio of 1:1 and printed together. Clones of the cells appeared 1 week after printing, suggesting cell proliferation and clonal expansion (Fig. 2A, confocal microscopy images). The co-cultured printed constructs intentionally had twice the cell density from the start compared to the control constructs with only iPSCs or iChons to maintain a comparable constant iPSC density due to the mortality of the iChons (Fig. 2B). The iChons control constructs showed a decreasing number of visible cells over time when analyzed by TPEF microscopy and were diminished after 3 weeks in culture (Fig. 2B, iChons, and Supplementary Fig. 6). In agreement with previous findings that the use of irradiation to make chondrocyte replication incompetent also causes apoptosis, all cells were dead within 25 days of irradiation^12^. Fluorescence *in situ* hybridization (FISH) analysis of the 3D-printed constructs after 5 weeks (Fig. 2C) further supported the disappearance of iChons over time. This observation implies that the iPSCs are the most likely source for cartilage matrix formation. The co-cultured printed protocol gave rise to the appearance of hyaline-like cartilaginous tissue at week 5, which was visualized by (i) Alcian blue-van Gieson staining of collagen, connective tissue and acidic polysaccharides, such as glycosaminoglycans in cartilage (blue), and (ii) Safranin-O staining, which stains cartilage red (Fig. 3B). Furthermore, areas with dark staining indicated hyaline cartilage tissue generation that was similar to stained native cartilage tissue and, in areas of increased Alcian blue-van Gieson staining, nuclei clusters with lacunae were seen (Fig. 3B, zoomed in, upper row). An increase in cell number was correlated with higher GAG production (Fig. 3B and C (TPEF, cells are yellow in 3C), corresponding areas in an unstained serial section are marked with red and green squares). While no significant difference in the SHG signal could be detected between the areas (Fig. 3C), the production of cartilage-specific collagen type II was evident by immunohistochemistry using a specific antibody for collagen type II (Fig. 3D, green; and Supplementary Fig. 7A) and was surprisingly more intense than the human control cartilage (Supplementary Fig. 7B), further suggesting that cartilage-like tissue had been generated after five weeks of differentiation. The immunohistochemistry results were important since the prints containing alginate, even without cells, gave a background staining with Alcian blue-van Gieson. The lack of significant difference in the SHG signal between the areas (Fig. 3C) could result from the fine collagen II fibrils generating such a weak SHG signal that their build-up disappears in the high background interference from cellulose. The sensitivity of this technique could be improved by using polarization sensitive SHG microscopy^25^. We next wanted to determine whether we could generate cartilage mimic tissue from iPSCs without using iChons. We succeeded in generating higher GAG production from iPSCs in an area (Fig. 4A and B) when the iPSC prints (60:40 NFC/A) were initially maintained in a conditioned DEF medium since single cell survival is dependent on factors produced from other iPSCs (Fig. 4C). Finally, we assessed the level of pluripotency protein OCT4 prior to and after printing (Fig. 5). OCT4 could be detected even in the presence of iChons, which have been shown to produce BMP2^12^, after 1 week of printing when maintained in the conditioned DEF medium. OCT4 signals were not detected after differentiation for 3 or 5 weeks (Fig. 5).

## Discussion

Even when we varied the bioink composition of NFC/A, we showed that the iPSCs remained in a pluripotent state (as determined by the OCT4 protein level). In particular, cells in the NFC/A 60/40 system were able to maintain a pluripotent phenotype after 3D bioprinting when using iPSC-conditioned medium, which probably helped the cells to sense that they were not isolated. The OCT4 protein (encoded by Pou5f1) was undetectable in the prints after 5 weeks of differentiation, which is important for implementing this technology in a clinical setting because pluripotency increases the risk of potential tumor formation. Hyaline-like cartilaginous tissue expressing collagen type II was observed in the histology sections of the 3D bioprints after five weeks when using the co-culturing with irradiated chondrocytes protocol and bioink composed of NFC/A 60/40. Moreover, an increase in cell number was observed in the cartilaginous tissue within the 3D-bioprinted constructs, thus highlighting the importance of cell quantity for cartilage production. We concluded that NFC/A bioinks were suitable for bioprinting iPSCs to support cartilage production in co-culture with irradiated chondrocytes. We noticed that after printing, viable cells increased in number over time, which was in agreement with previous observations with other cell types that human chondrocytes bioprinted in noncytotoxic, nanocellulose-based bioink exhibited cell viabilities of 73% and 86% after 1 and 7 days of 3D culture, respectively^26, 27^. The positive co-culture effect supported earlier findings that irradiated chondrocytes would stimulate iPSC differentiation by cell-to-cell contact combined with local inherent growth factors^8, 28^. BMP2 has previously been determined to be one of the growth factors secreted by irradiated chondrocytes^12^. By including this morphogene in the chondrogenic differentiation medium, we could enhance the co-culture protocol and detect the differentiation of iPSCs without co-culture. GDF5, another growth factor that we included in the differentiation medium, also belongs to the bone morphogene protein (BMP) family. Determining which growth factors to include or exclude, along with the timing and concentration of growth factors, is key to further improving these types of protocols. In summary, the identification of a protocol using NFC/A that after 3D bioprinting, directed iPSCs into chondrogenic commitment could permit further delineation of the molecular pathway to optimize and improve articular cartilage tissue generation. We believe that this research will help bring forward 3D bioprinting with iPSCs as a future treatment to repair damaged cartilage in joints.

## Methods

A bioink composed of nanocellulose and alginate was prepared for 3D bioprinting. Different fractions of nanocellulose and alginate were prepared for cell encapsulation, and the viability of the cells was evaluated after printing. Cell morphology and distribution were analyzed by nonlinear microscopy. iPSCs were mixed with irradiated chondrocytes (to acquire a chondrocyte-free print, the chondrocytes were irradiated (25 Gray) prior to mixing) and 3D bioprinted to direct differentiation towards cartilage. Up to 6 weeks after printing, the 3D bioprinted constructs were sectioned and stained with Alcian blue-van Gieson.

### Fabrication of bioinks

The bioinks were prepared as described previously^26^. Briefly, nanofibrillated cellulose (NFC) was produced using mechanical refinement and enzymatic treatment^29^. NFC was sterilized using electron irradiation at 25 kGy. Sterile alginate (150–250 kDa; FMC Biopolymers, Norway) with ≥60% α-1-guluronic acid was used for the bioink. The composition of the bioink was calculated by the weight percent of the complete hydrogel. To obtain the physiological osmolarity, 4.6% mannitol was added to the hydrogel solution^30^.

Corgel® Biohydrogel, a hyaluronan-based hydrogel (HA) with 5% tyramine substitution, was purchased from Lifecore Biomedical (MN, USA). A 5% (volume %) final hydrogel of HA in nanocellulose was produced for the encapsulation or printing of iPSCs and/or irradiated chondrocytes. After the constructs were formed or printed, a solution of 0.001% (v/v) H_2_O_2_ in water was used for crosslinking for 5 minutes.

### Culture of human iPSCs and irradiated chondrocytes

We previously generated iPSC lines from surplus chondrocytes using mRNA-based reprogramming^6^. The A2B iPSC line was maintained under feeder-free conditions in Cellartis DEF-CS™ (TaKaRa ClonTech, Sweden). This iPSC line was karyotype-tested, was normal even at late passages, was pluripotent with regards to the expression of pluripotency markers and was able to differentiate into all germ layers^6^ (Supplementary Fig. 8). This line was also shown to be superior in the differentiation protocol to generate articular cartilage matrix in 3D pellets and was used for 3D printing in subsequent experiments. In addition, iPSC-conditioned DEF medium from confluent clone A2B iPSCs was used after printing since increased survival has been noticed for single cells in a conditioned medium. For co-culture conditions, human surplus chondrocytes were irradiated (iChons) before being mixed with iPSCs to prevent the proliferation of the chondrocytes. The cell number was counted in a nucleocounter NC-200^TM^ using Via1-Casettes^TM^ (ChemoMetec, Denmark).

### Three-dimensional bioprinting

The final concentration of iPSCs and/or irradiated chondrocytes (iChons) was 20 million cells per ml of bioink. A *3D Discovery* (regenHu, Switzerland) 3D bioprinter was used with a 300-µm nozzle. The grid construct was designed using the BioCAD software (Biomedical Modeling Inc, USA) and was printed using the 3D Discovery HMI software. Printing parameters were set to 10–20 mm/s feed rate, 20–30 kPa pressure, and 0.05–0.07 mm dosing distance. Prior to printing, the apparatus was sterilized using 70% ethanol, and filtered air (0.22 µm) was used to reduce the risk of contamination. Printing was performed at room temperature in an open area within a clean room. The printing occurred at ambient temperature and humidity, and six-layer grids were printed into 24-well plates. The grids were 7 by 7 mm and 1.2 mm thick (Supplementary Fig. 4). Immediately after printing, the NFC/A constructs were crosslinked in 100 mM CaCl_2_ solution for 5 minutes, and NFC/HA constructs were crosslinked with a water solution of 0.001% (v/v) H_2_O_2_ for 5 minutes (iPSCs controls, Supplementary Fig. 1). The HA crosslinking occurred at the substituted functional tyramine group, where a covalent bond at the tyramine carbon ring was formed. After crosslinking, the constructs were briefly rinsed in the culture medium, which was replaced with fresh medium. The constructs were thereafter transferred to an incubator held at 37 °C and 5% CO_2_. The rheological properties, shape fidelity and mechanical strength of the 60/40 and 80/20 bioinks have been previously tested^26^.

### Directed chondrogenic differentiation of iPSCs in 3D-printed constructs

Equal numbers of iPSCs were mixed with iChons to give a final concentration of 20 million cells/ml in the bioink, and the printed constructs were maintained at 37 °C and 90% humidity in 5% CO_2_. After 7 days of culture in the DEF CS™ (TaKaRa ClonTech, Sweden) pluripotent medium mixed with an equal volume of conditioned DEF-CS medium (conditioned DEF medium was taken from 80% confluent pluripotent iPSCs after 24 hours of culture, sterile filtered, and fresh growth factors (GF1, GF2 and GF3; TaKaRa ClonTech, Sweden) were added), the medium was changed every day. Cells were then initiated to differentiate by changing the medium to a defined chondrogenic medium (high-glucose Dulbecco’s modified Eagle’s medium; PAA Laboratories) supplemented with 5.0 μg/ml linoleic acid solution (Sigma-Aldrich), 1x ITS-G premix (10 mg/l insulin, 5.5 mg/l transferrin, 6.7 μg/l selenious acid; Life Technologies), 0.11g/l sodium pyruvate, 1.0 mg/ml human serum albumin (Equitech-Bio, TX, USA), 10 ng/ml TGFβ1 (R&D Systems, Abingdon, UK), 10 ng/ml GDF5, 10 ng/ml BMP2, 100 nM dexamethasone (Sigma-Aldrich), 80 μM L-ascorbic acid (Sigma-Aldrich), and 1x penicillin/streptomycin (PEST; PAA Laboratories). The medium was changed three times a week. Relevant control cultures with only printed irradiated chondrocytes were kept throughout the differentiation protocol.

### Histological preparations

Samples were rinsed twice with PBS containing CaCl_2_ before fixation with Histofix (5% paraformaldehyde; HistoLab Products AB, Sweden) for 20 minutes. CaCl_2_ prevented the prints from disintegrating. Afterwards, the samples were rinsed twice with PBS before being stored in 100 mM CaCl_2_ for transport to HistoLab (Gothenburg, Sweden) for paraffin embedding, slicing (10-μm sections), and staining with Alcian blue and van Gieson’s dye for glycosaminoglycans, Safranin O for cartilage-like extracellular matrix production, and hematoxylin and eosin for nuclei and matrix components. An upright Nikon Eclipse 90i microscope was used to obtain images of the histology slices.

FISH analyses for chromosomes X and Y were performed on histology sections at the Department of Clinical Chemistry at Sahlgrenska University Hospital.

### Collagen II immunohistochemical analysis

Histology sections of the 3D prints were deparaffinized, rehydrated and treated with 8,000 U/ml of hyaluronidase (Sigma-Aldrich, St. Louis, MO) in PBS for 60 minutes at 37 °C. The sections were blocked with 3% bovine serum albumin (Sigma-Aldrich) in PBS and incubated with a primary mouse anti-human collagen II antibody, diluted 1:150 (MP Biomedicals Europe, Illkirch, France), at 4 °C overnight, followed by a 2 hour incubation with a secondary antibody at room temperature in darkness using goat anti-mouse Alexa Fluor 488 diluted 1:400 (A11017; Life Technologies). Samples were mounted using ProLongGold Antifade that included DAPI (4,6-diamidino-2-phenylindole; Life Technologies) to visualize nuclei. Samples were observed using a Nikon Eclipse Ti-U fluorescence microscope.

### Pou5f1 (Oct4) immunohistochemical analysis

Samples stained for Oct4 were (i) iPSCs seeded on coat 1 (TaKaRa ClonTech, Sweden) and exposed to NFC/A or NFC/HA for 2 days in the DEF medium (TaKaRa ClonTech, Sweden), and (ii) serial sections from the 3D prints that were first deparaffinized. The primary antibody used was rabbit anti-Oct4 diluted 1:400 (C30A3; Cell Signaling Technology, Danvers, MA, http://www.cellsignal.com). The secondary antibody used was goat anti-rabbit Alexa Fluor 546 diluted 1:400 (A11071; Life Technologies).

### Microscopy

Microscopy images were taken of samples with (i) live/dead staining on a wide-field fluorescence microscope; (ii) actin/nuclear staining on a confocal microscope; (iii) no labels on a nonlinear optical microscope, which has been described previously^31^; and (iv) bright-field and fluorescence images on a Nikon Eclipse Ti-U with an attached Andor Zyla camera.

Label-free imaging of cellular intrinsic fluorescence and matrix anisotropy was performed using two-photon excitation fluorescence (TPEF) and second-harmonic generation (SHG). A diode-pumped solid-state laser (Nd:vanadate, 10 W) was used to generate two-picosecond pulsed beams: 1064 nm and 532 nm (7 ps, 76 MHz). The 532 nm beam was guided into an optical parametric oscillator (Levante Emerald OPO, APE Berlin, 690–900 nm) to generate a beam at 817 nm. Laser beams were directed onto samples mounted on an inverted microscope (Eclipse TE2000-E with a C2 confocal microscope scanning head, Nikon) with a 40x oil-immersion objective (Nikon Plan Fluor, NA 1.30). TPEF and SHG signals were obtained with 405 ± 10 nm and 609 ± 57 nm optical-density filters, respectively. Single photon counters from Becker & Hickl Gmbh were used to detect TPEF and SHG simultaneously. Image analysis and 3D rendering were performed with ImageJ (National Institutes of Health, USA).

### RNA extraction and RT PCR (Reverse transcription-polymerase chain reaction)

Samples were frozen at −80 °C at various time points for RNA extraction. Lysis of the construct was performed with RLT buffer from the Qiagen Mini-Kit and Matrix Lysis D (MP Biologics), which was shaken at 25 Hz for 2 minutes on a Qiagen Tissue Lyser. The lysate was then used for RNA extraction following the standard protocol of a Qiagen Mini Kit. The RNA concentration and quality were obtained immediately after extraction using the NanoDrop 2000 (Thermo Fisher). For cDNA synthesis and quantitative reverse transcriptionase polymerase chain reaction (PCR), all reagents, instruments, and software were purchased from Applied Biosystems (Life Technologies). The cDNA was prepared from total RNA using a High-Capacity cDNA Reverse Transcriptase Kit with random hexamers and RNase Inhibitor on a 2720 Thermal Cycler. All samples were analyzed in duplicate on the 7900HT instrument using TaqMan Gene Expression Master Mix. The following human TaqMan gene expression assays were used: SOX9 (Hs00165814_m1), COL2A1, splice variant (Hs01064869_m1), and ACAN (Hs00153936_m1). CREBBP (Hs00231733_m1) was used as a reference gene. All samples were treated with RNase-Free DNase (Qiagen Gmbh, Germany) to avoid genomic DNA contamination. The fold change for each sample was calculated using the 2−ΔΔCT method^32, 33^, and the expression level was calculated relative to an in-house calibrator.

## Electronic supplementary material

SUPPLEMENTARY INFO NGUYEN ET AL
