# Supplementary material for: Cartilage Tissue Engineering by the 3D Bioprinting of iPS Cells in a Nanocellulose/Alginate Bioink
Source: Sci Rep. 2017 Apr 6;7:658. doi: 10.1038/s41598-017-00690-y (PMC5428803; doi:10.1038/s41598-017-00690-y)
Supplement: Supplementary file 1 — SUPPLEMENTARY INFO NGUYEN ET AL [file 41598_2017_690_MOESM1_ESM.pdf]

Supplementary Information

Cartilage Tissue Engineering by the 3D Bioprinting of iPS Cells in a Nanocellulose/Alginate Bioink

Duong Nguyen[2,3], Daniel A Hägg[1], Alma Forsman[3], Josefine Ekholm[3], Puwapong Nimkingratana[3], Camilla Brantsing[3], Theodoros Kalogeropoulos[1], Samantha Zaunz[1], Sebastian Concaro[4], Mats Brittberg[4], Anders Lindahl[3], Paul Gatenholm[1,5], Annika Enejder[2] and Stina Simonsson[3*]

[1] 3D Bioprinting Center, Dept. of Chemistry and Chemical Engineering, Chalmers University of Technology, Gothenburg, Sweden

[2] Chemical Biology, Dept. of Biology and Biological Engineering, Chalmers University of Technology, Gothenburg, Sweden

[3] Institute of Biomedicine at Sahlgrenska Academy, Department of Clinical Chemistry and Transfusion Medicine, University of Gothenburg, Sweden

[4] Cartilage Repair Unit, University of Gothenburg, Region Halland Orthopaedics, Kungsbacka Hospital, Kungsbacka, Sweden

[5] Wallenberg Wood Science Center, Chalmers University of Technology, Gothenburg, Sweden

*Correspondence to stina.simonsson@gu.se

Supplementary Figure 1

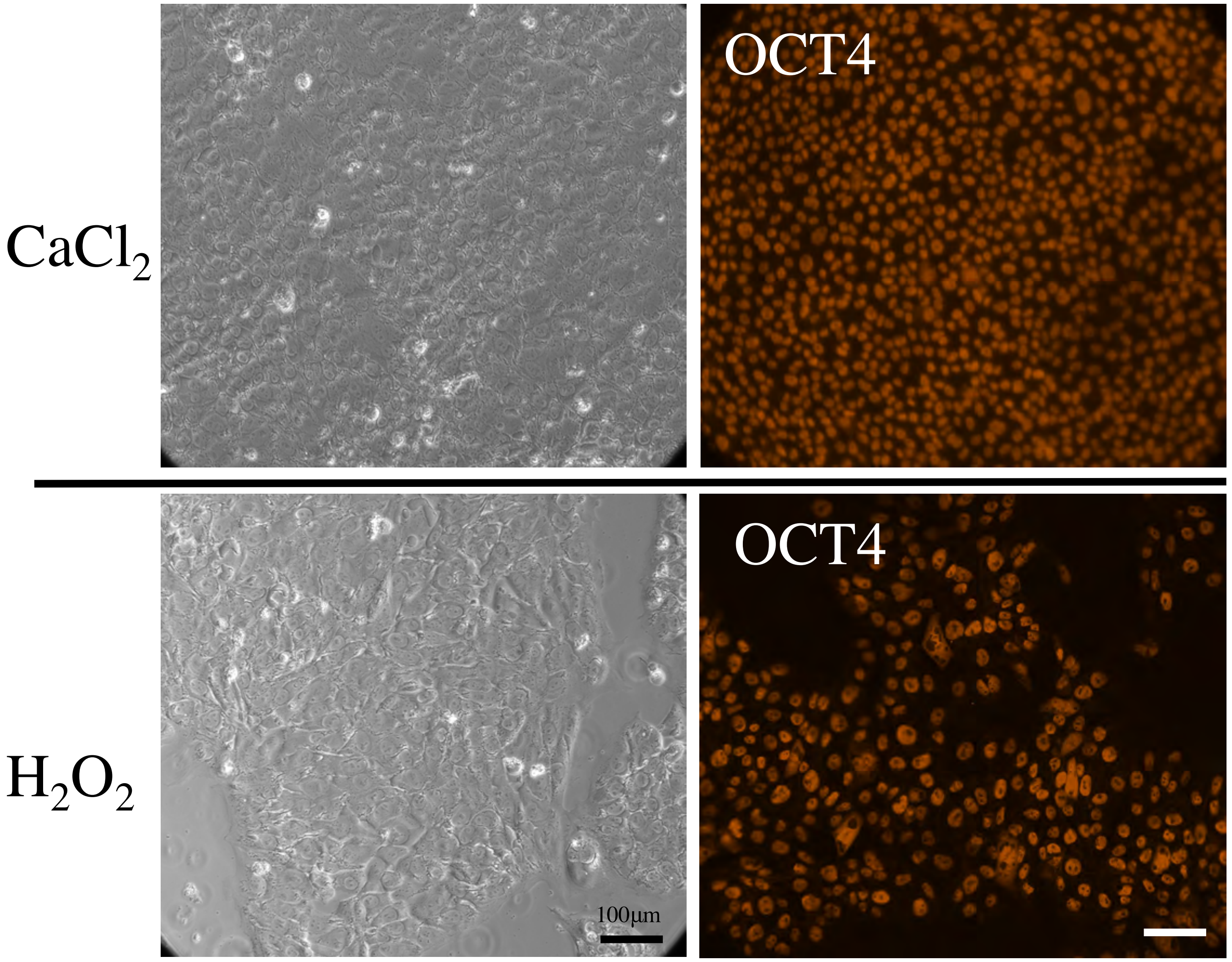

Supplementary Figure 2

Cluster sizes for Alginate and HA ink

Growth	Alginate (4x)	HA ink (4x)
Day 2	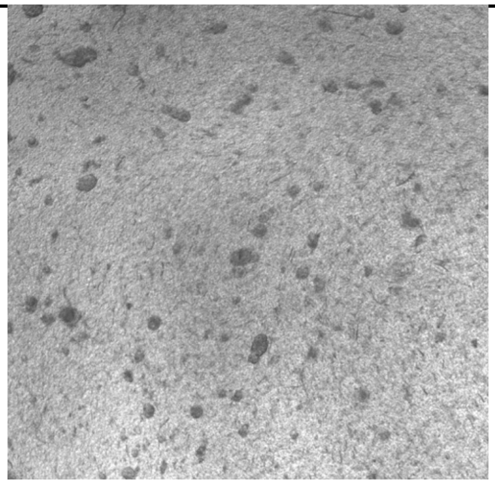	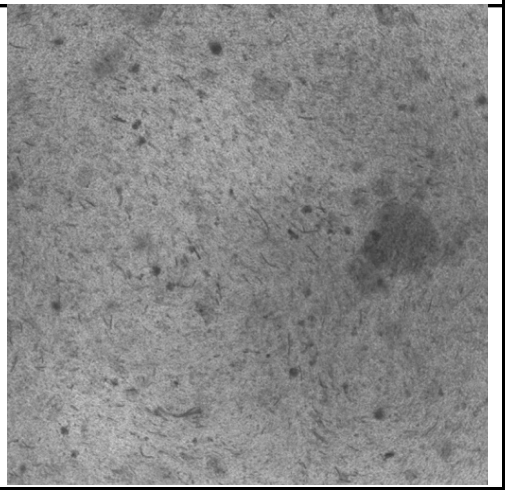
Cluster Diameter	93.4+/- 20μm	53.3+/- 12.2μm
Day 6	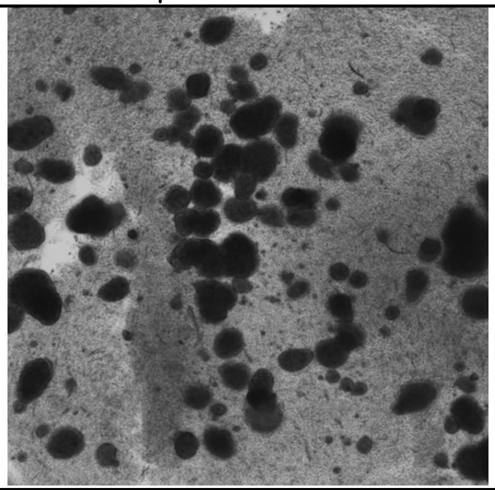	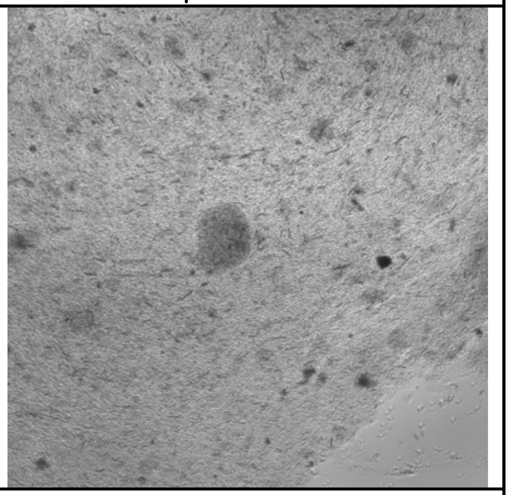
Cluster Diameter	183.3+/- 32.2μm	38.9+/- 9.7μm
Day 14	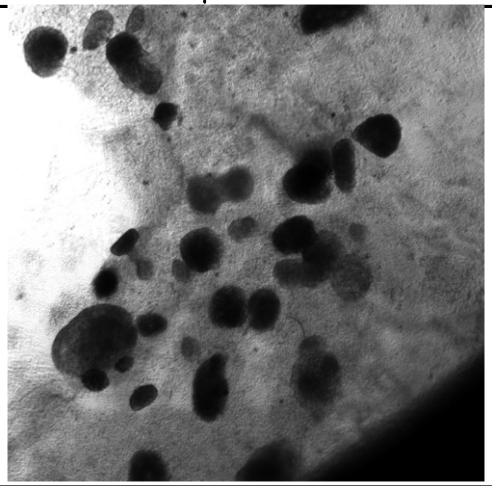	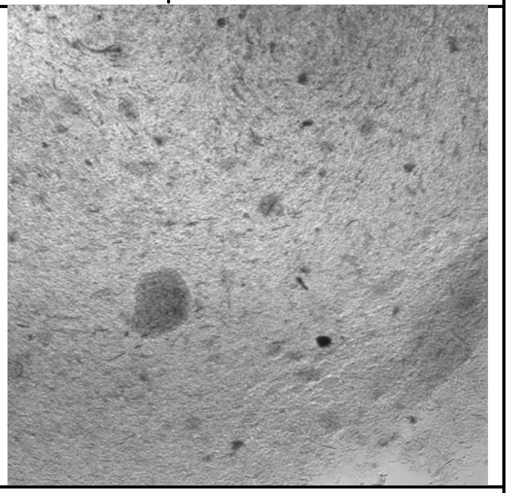
Cluster Diameter	249.1+/- 79.3μm	42.8+/- 10.9μm

Supplementary Figure 3

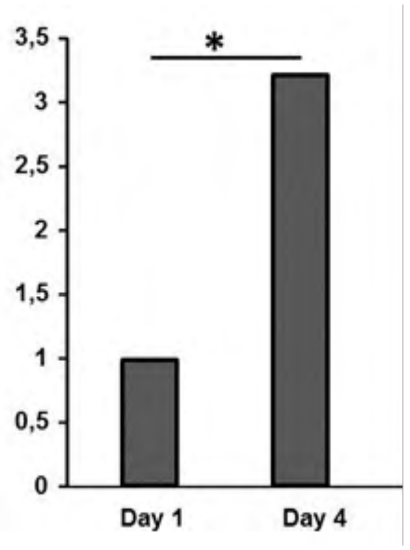

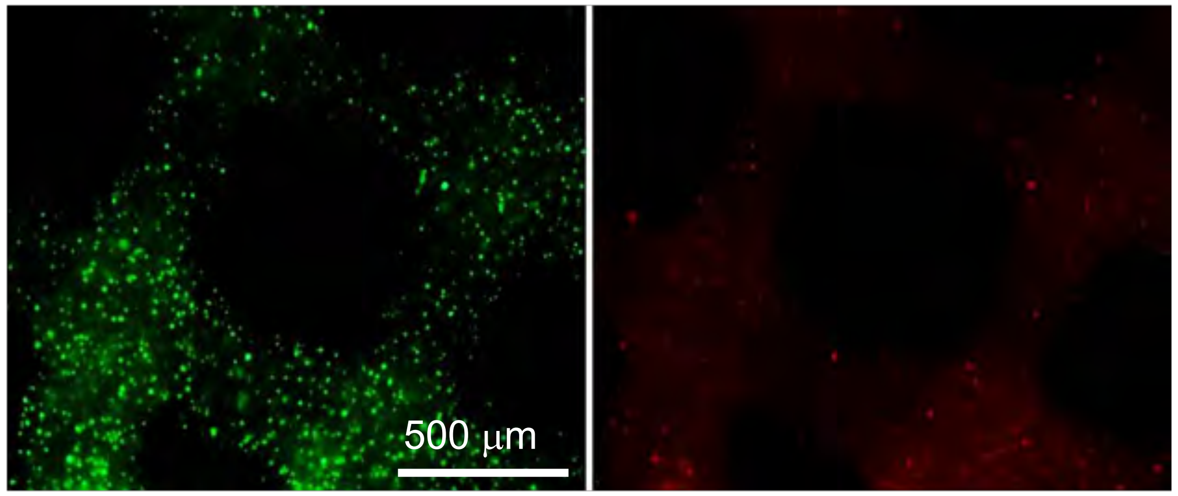

Supplementary Figure 4

A

NFC/A Bioink

3% | 97% Aqueous solution

3% Dry content: NFC + Alginate

NFC/A 80/20: 2.4% NFC + 0.6% Alginate

NFC/A 60/40: 1.8% NFC + 1.2% Alginate

NFC/HA Bioink

5 volume % HA | 95 volume % NFC

B

NFC/A 80/20 | NFC/A 60/40

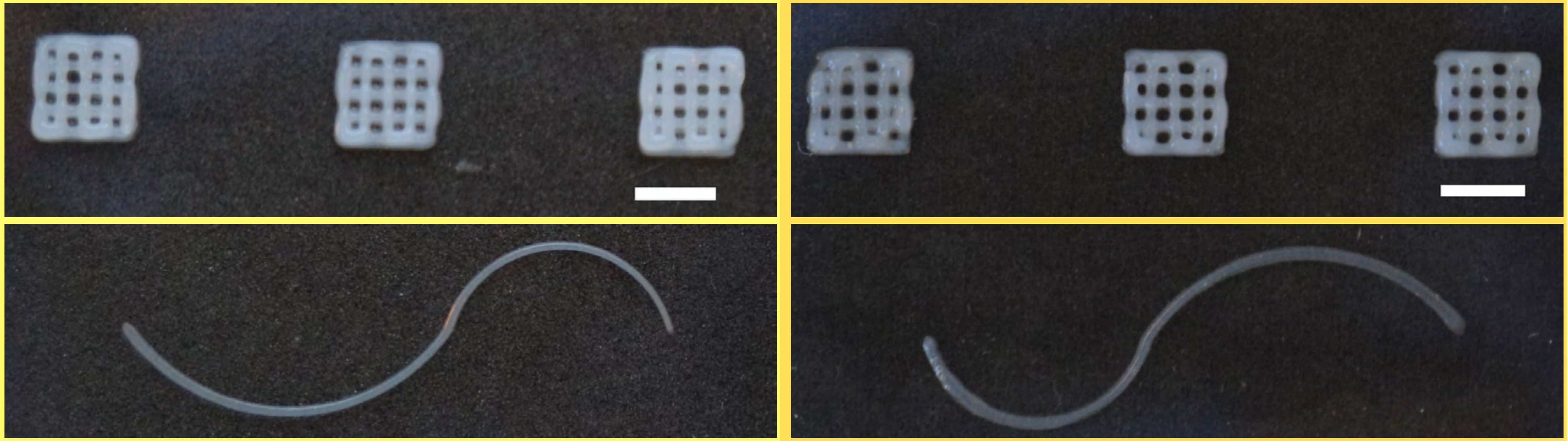

C

NFC/A 80/20		NFC/A 60/40	
iPSCs<br>10 million/ml bioink	iPSCs + iChon<br>20 million/ml bioink	iPSCs<br>10 million/ml bioink	iPSCs + iChon<br>20 million/ml bioink

NFC/HA	
iPSCs<br>10 million/ml bioink	iPSCs + iChon<br>20 million/ml bioink

Supplementary Figure 5

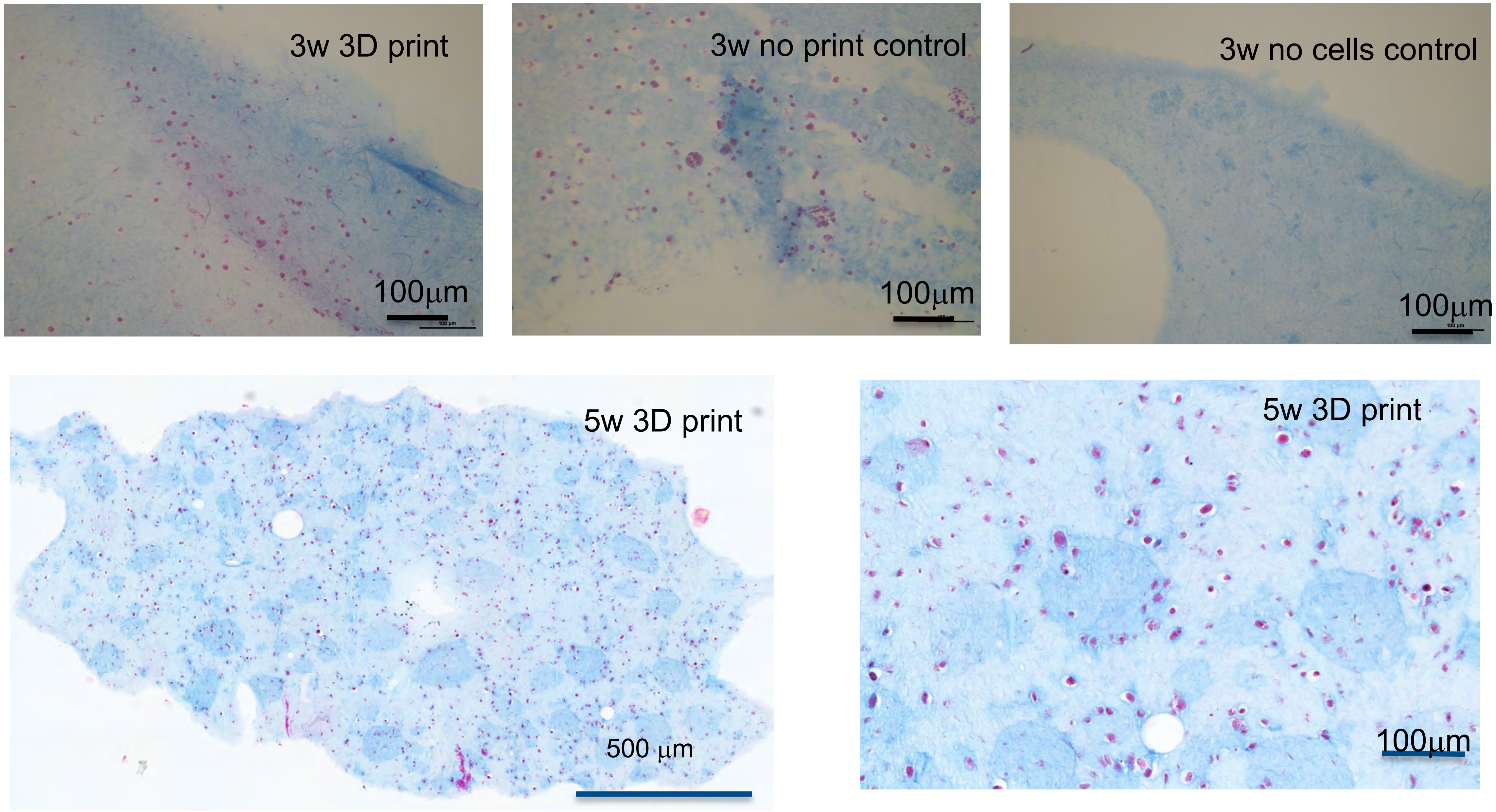

Supplementary Figure 6

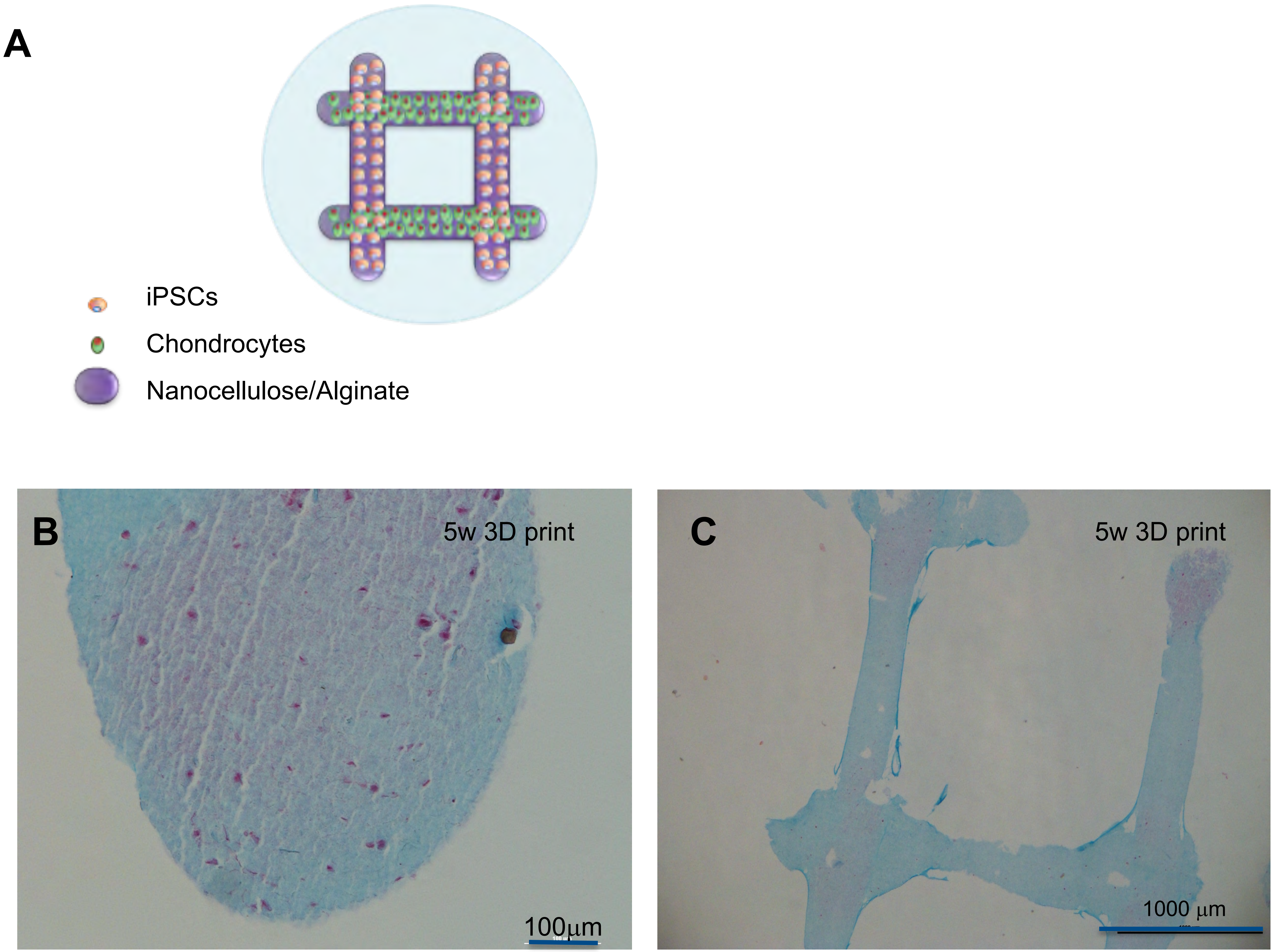

Supplementary Figure 7

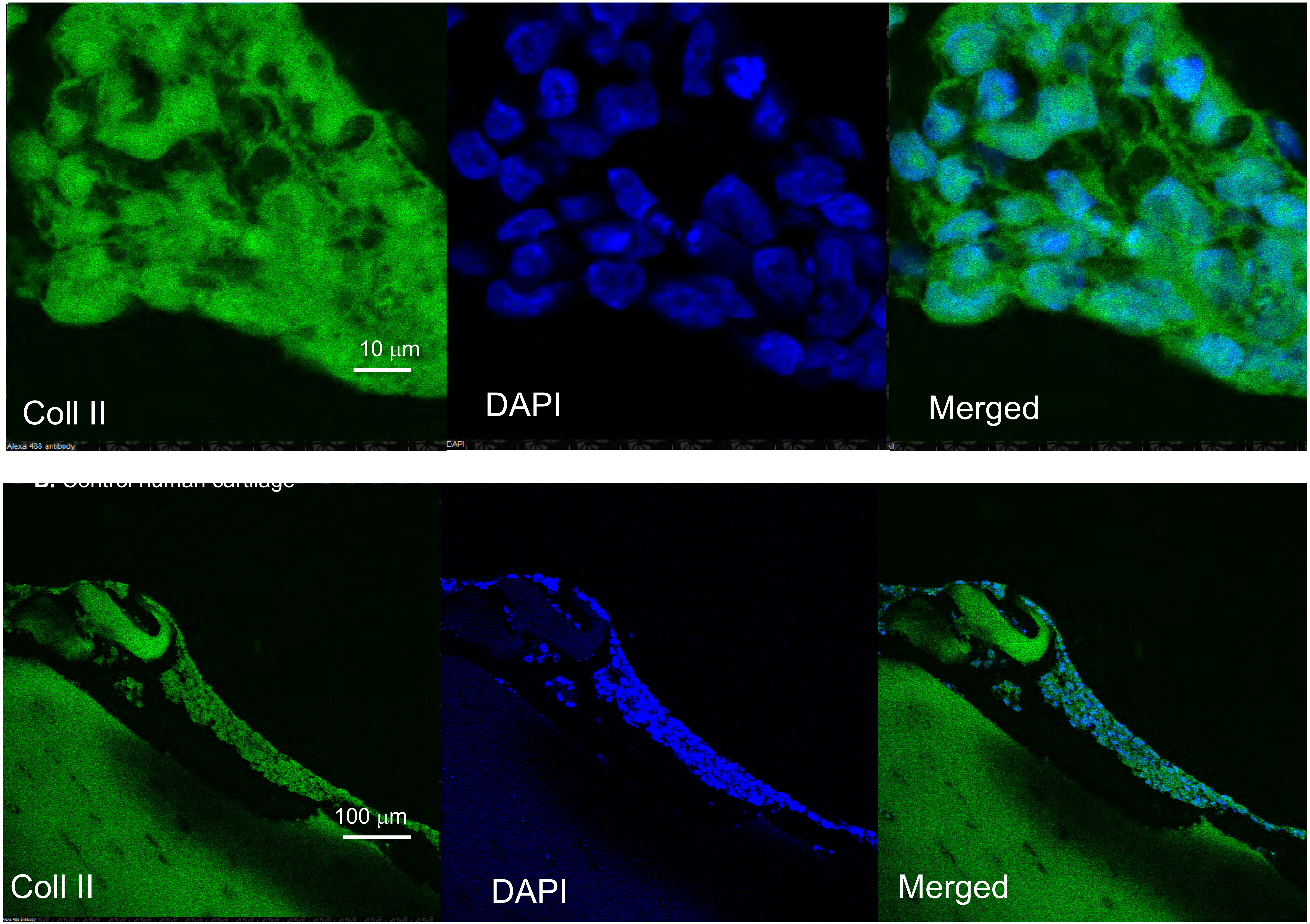

Supplementary Figure 8

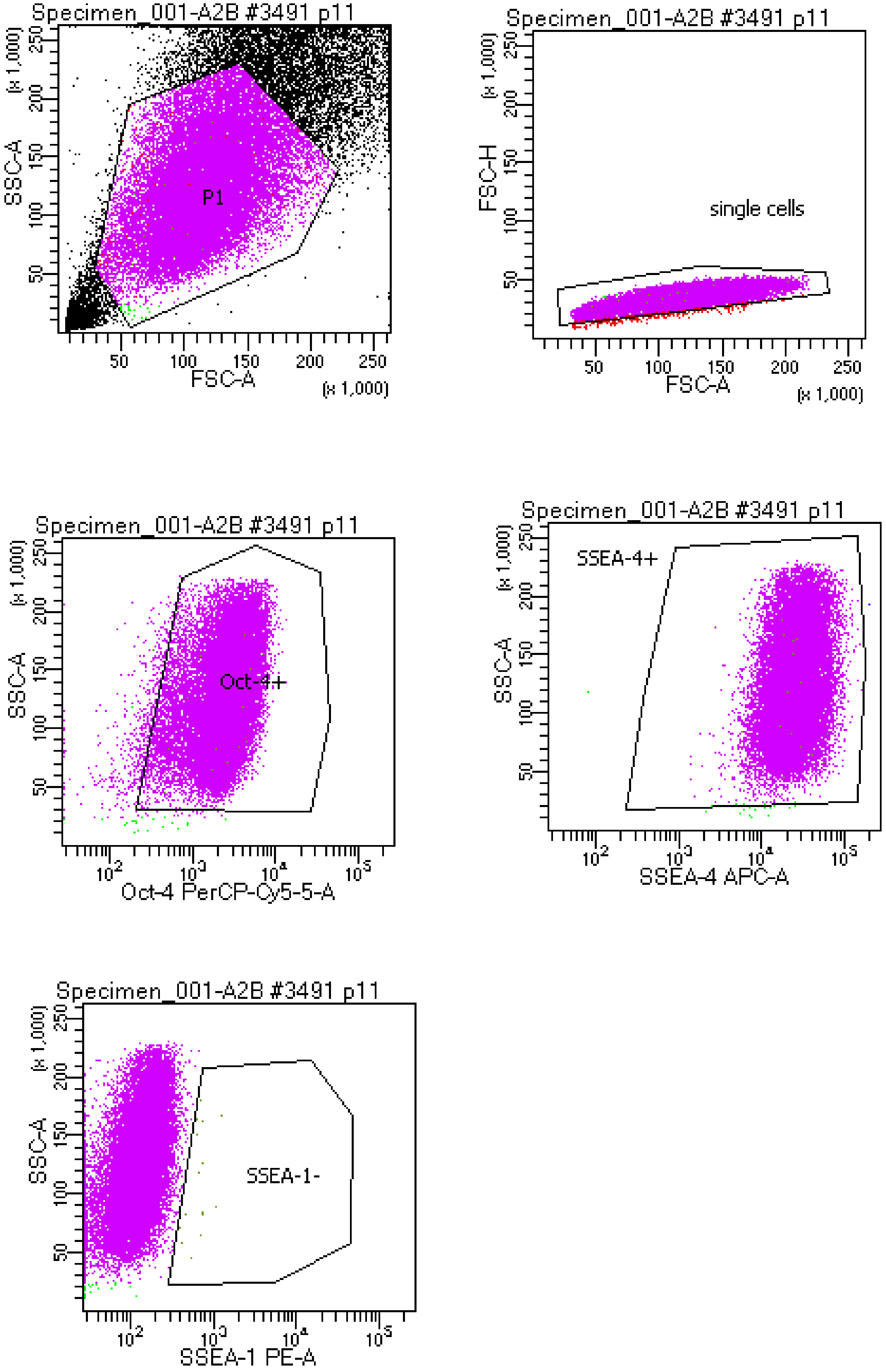

Supplementary Figure 9

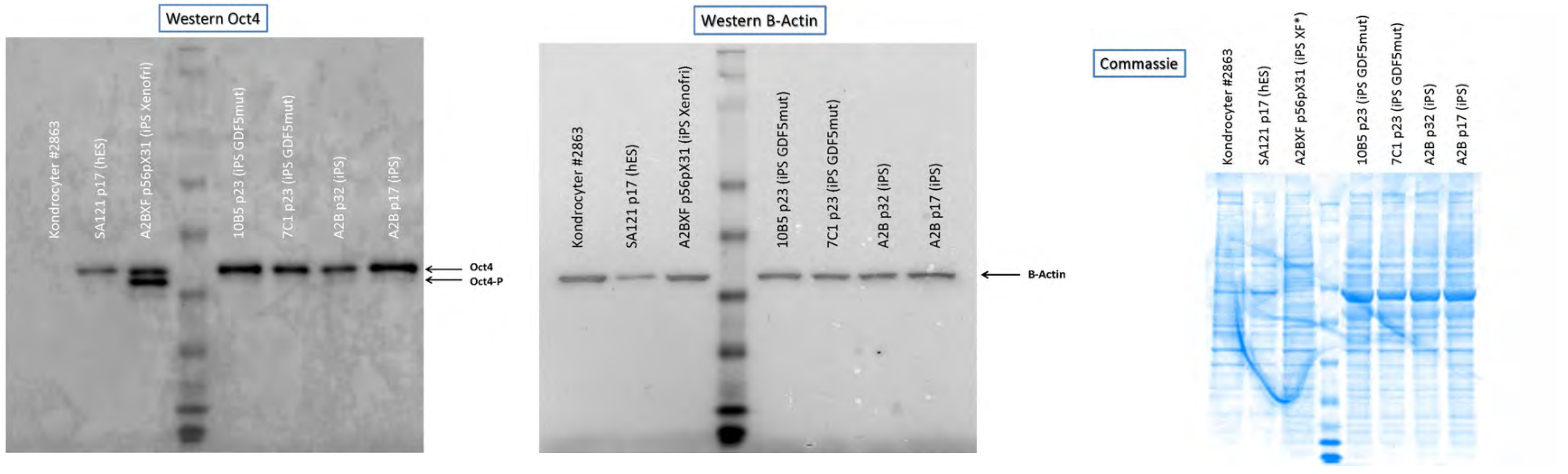

Supplementary Figure 10

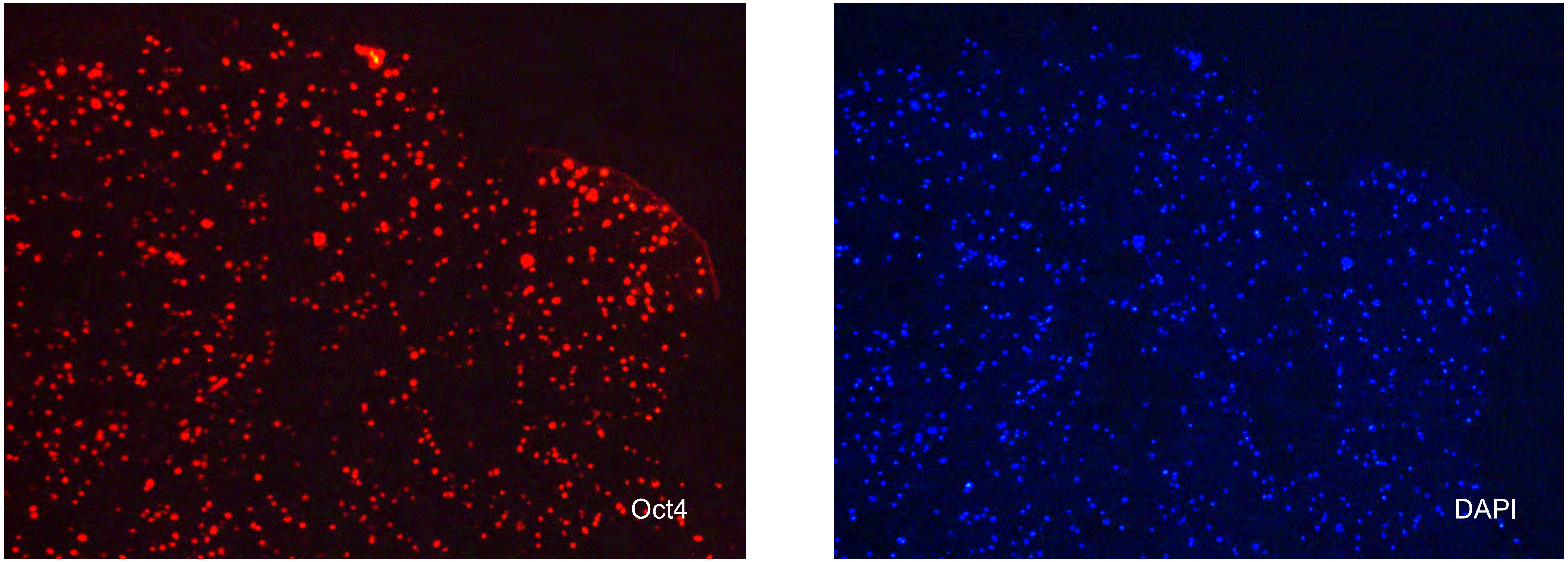

Supplementary table 1

Cluster sizes for Fig1B

	NFC/NA 60/40 (4x)	NFC/A 80/20 (4x)	NFC/HA
Cluster Diameter	213.3+/- 74.3µm	108.9+/- 41.9µm	38.9+/- 9.7µm

Supplementary Figure Legends

Supplementary Figure 1. $CaCl_2$ or H_2O_2 effects on iPSCs. Bright-field images (left) and Oct4 immunohistochemistry (right) after 5 minutes of treatment with the crosslinking reagents. Without the addition of the hyaluronic hydrogel, the crosslinking reaction did not occur; hence, the Oct4 expression levels of iPSCs were maintained.

Supplementary Figure 2. Cluster sizes for alginate and hyaluronic hydrogels. Alginate and hyaluronic hydrogels were used to encapsulate the iPSCs to demonstrate cell compatibility. The alginate hydrogel supported proliferation within clusters, while the hyaluronic hydrogel showed no cell clusters or proliferation after 14 days of culture. Measurements of the clusters (n=20) were used to demonstrate the growth of the clusters.

Supplementary Figure 3. Cell proliferation after 3D bioprinting with iPSCs. (**Left**) Cell proliferation was analyzed by performing an MTS assay (Promega). Four days after printing, the MTS signal increased three-fold compared to that at day one, indicating that the cells proliferated inside the 3D-bioprinted scaffolds. *p=0.000024 (**Right**) Live/dead (green/red; left/right) staining five days after printing showed that the viability of the iPSCs was good.

Supplementary Figure 4. Hydrogel composition and printability. (A) A detailed schematic of the two different NFC/A compositions (NFC/A 80/20 and NFC/A 60/40). (B) Replicate prints of NFC/A 80/20 and NFC/A 60/40, and an S-test used to demonstrate the printing resolution of each bioink. Scale bar=10 mm.

Supplementary Figure 5. Three-dimensional-bioprinted iPSCs in NFC/HA bioink. Alcian blue and van Gieson’s dye staining of (A) the bioprinted iPSC in NFC/HA, (B) the

iPSCs in the HA control, and (C) the no cell control of NFC/HA after 3 weeks of culture. (D-E) Alcian blue and van Gieson’s dye staining of the bioprinted iPSCs in NFC/HA after 5 weeks of culture.

Supplementary Figure 6. Three-dimensional-bioprinted iPSCs in NFC/A 60/40 bioink. (A) Schematic picture of the 3D printing experiment in B and C with the iPSCs and irradiated chondrocytes (iChon). The different cell types were printed perpendicular to each other in different lanes. (B-C) Positive red glycan staining only at the intersections where iPSCs and chondrocytes were overlapping after 5 weeks of differentiation.

Supplementary Figure 7. Collagen type II immunohistochemistry. The positive collagen type II staining (green; DAPI, blue) was within the cluster of cells in the 3D-printed iPSCs/iChon after 5 weeks of differentiation. Human cartilage was used as the positive control (lower row). The scale bars in the confocal images represent 10 and 100 μm in the upper and lower rows, respectively.

Supplementary Figure 8. Fluorescence-activated cell sorting (FACS) of the human iPSC line A2B are Oct4 and SSEA4 positive and SSEA1 negative. These results are in accordance with what has been reported for human pluripotent stem cell lines.

Supplementary Figure 9. Oct4 Western blot shows pluripotency of iPS line A2B. Complete Western blots and corresponding commassie stained gel shown in Figure 5A.

Supplementary Figure 10. Complete Oct4 immunohistochemistry of 3D-printed iPSCs/iChon after 1 week post printing shown in Figure 5B.

Supplementary Table I. Cluster sizes for Figure 1B. The cell clusters in Figure 1 were measured to demonstrate the differences in cluster proliferation within the three different bioinks.
